# The Use of Vitamin K2 in Patients With Parkinson's Disease and Mitochondrial Dysfunction (PD-K2): A Theranostic Pilot Study in a Placebo-Controlled Parallel Group Design

**DOI:** 10.3389/fneur.2020.592104

**Published:** 2021-01-11

**Authors:** Jannik Prasuhn, Meike Kasten, Melissa Vos, Inke R. König, Sebastian M. Schmid, Britta Wilms, Christine Klein, Norbert Brüggemann

**Affiliations:** ^1^Institute of Neurogenetics, University of Lübeck, Lübeck, Germany; ^2^Department of Neurology, University Medical Center Schleswig-Holstein, Lübeck, Germany; ^3^Department of Psychiatry and Psychotherapy, University Medical Center Schleswig-Holstein, Lübeck, Germany; ^4^Institute of Medical Biometry and Statistics, University of Lübeck, Lübeck, Germany; ^5^Institute of Endocrinology and Diabetes, University of Lübeck, Lübeck, Germany; ^6^German Center for Diabetes Research (DZD), Neuherberg, Germany

**Keywords:** Parkinson's disease, treatments, vitamin K2, Parkin (PARK2), PINK1

## Abstract

**Background:** Despite rapid advances in research on Parkinson's disease (PD), in particular in the elucidation of genetic contributions, no disease-modifying therapy has become available to date.

**Objectives:** In the proposed project, we aim to investigate the potential effects of vitamin K2 (long-chain menaquinone 7, MK-7) in genetically determined PD with mitochondrial dysfunction.

**Methods:** A total of 130 study participants (26 biallelic *Parkin*/*PINK1* mutation carriers, 52 sporadic PD patients, and 52 healthy controls) will receive the trial medication (MK-7 or placebo for 1 week). 31P-Magnetic resonance spectroscopy imaging of the forebrain and basal ganglia (31P-MRSI, primary endpoint) as well as other advanced neuroimaging methods, clinical assessment, including quantitative movement analysis, and biomarker sampling will be applied pre- and post-intervention.

**Innovation:** The proposed project is highly translational as it builds on compelling mechanistic data from animal studies as well as on a small preliminary data set in humans. Patients are selected based on their mutation-related mitochondrial dysfunction and compared to disease and a healthy control group in a personalized medicine approach. We will further investigate how neuroimaging and blood-derived biomarkers can predict individual treatment response in sporadic PD.

**Clinical trial registration:** This study was registered at the German Clinical Trial Registry (DRKS, DRKS00019932) on the 19th of December 2019.

## Introduction

### Background and Rationale

**Figure 1:**
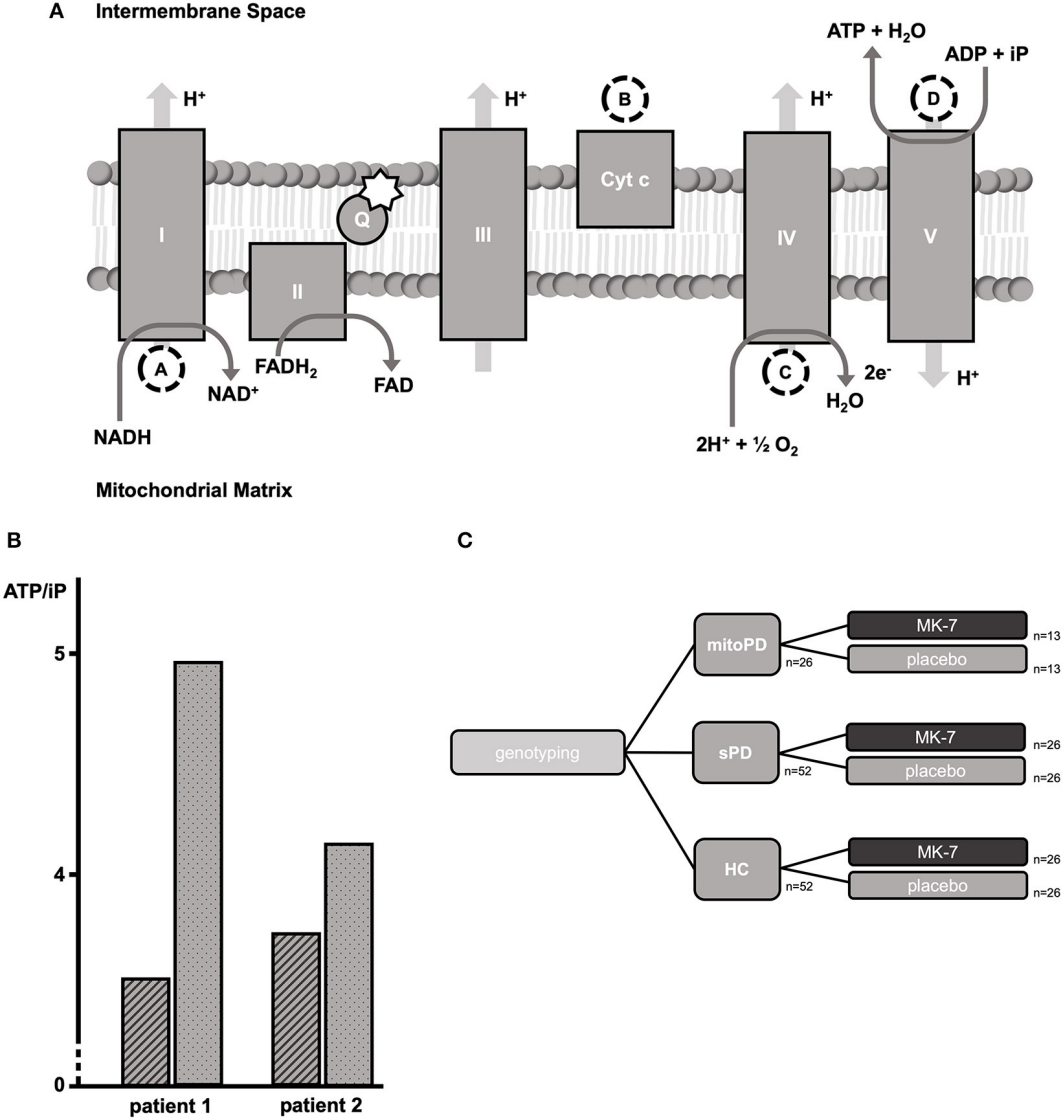
Neuroimaging approaches for investigating mitochondrial bioenergetics. The figure shows a simplified scheme of the electron transport chain (ETC). The dashed circles indicate which metabolic steps can be addressed via neuroimaging approaches. The star indicates the proposed method of action of MK-7 as an additional electron carrier within the Q cycle (8). We hypothesize that metabolic steps following the Q cycle might be of high predictive value for monitoring therapeutic response. **(A)** Changes in the oxidation of Cyt c will be measured as a chromophore via bNIRS (11, 12). **(B)** Measurements of the MRO_2_C and OEF will be measured by a combination of BOLD and ASL imaging (13, 14). **(C)** We will analyze the ATP/iP ratio with 31P-MRSI. The role of 1H-MRSI is not shown in the figure. We will utilize 1H-MRSI, however, to measure lactate and NAA. Lactate serves as a surrogate marker of anaerobic glycolysis as an alternative metabolic pathway if ATP production by oxidative phosphorylation is insufficient. NAA serves as a surrogate marker of mitochondrial membrane integrity (15). I, Complex I, NADH:ubiquinone oxidoreductase, NADH-CoQ reductase, or NADH dehydrogenase, 1H-MRSI: Proton Magnetic Resonance Spectroscopy Imaging; II, Complex II, succinate dehydrogenase or succinate-CoQ reductase; III, Complex III, cytochrome bc1 complex or CoQH2-cytochrome c reductase; IV, Complex IV, cytochrome c oxidase; V, Complex V, F_O_F_1_-ATPase; 31P-MRSI, 31-Phosphorus Magnetic Resonance Spectroscopy Imaging; ADP, adenosine diphosphate; ASL, arterial spin labeling; ATP, adenosine triphosphate; bNIRS, broadband near-infrared spectroscopy; BOLD, blood oxygen dependent imaging; Cyt c, cytochrome c; e^−^, electron; FADH_2_/FAD, flavine adenine dinucleotide; H^+^, proton; H_2_O, water; iP, inorganic phosphate; MK-7, long-chain menaquinone 7, vitamin K2; MRO_2_C, metabolic rate of oxygen consumption; NAA, N-acetylaspartate; NAD^+^/NADH, nicotinamide dinucleotide; O_2_, molecular oxygen; OEF, oxygen extraction fraction; Q, Q cycle, quinone cycle. Created with Biorender.com.

**Figure 2:**
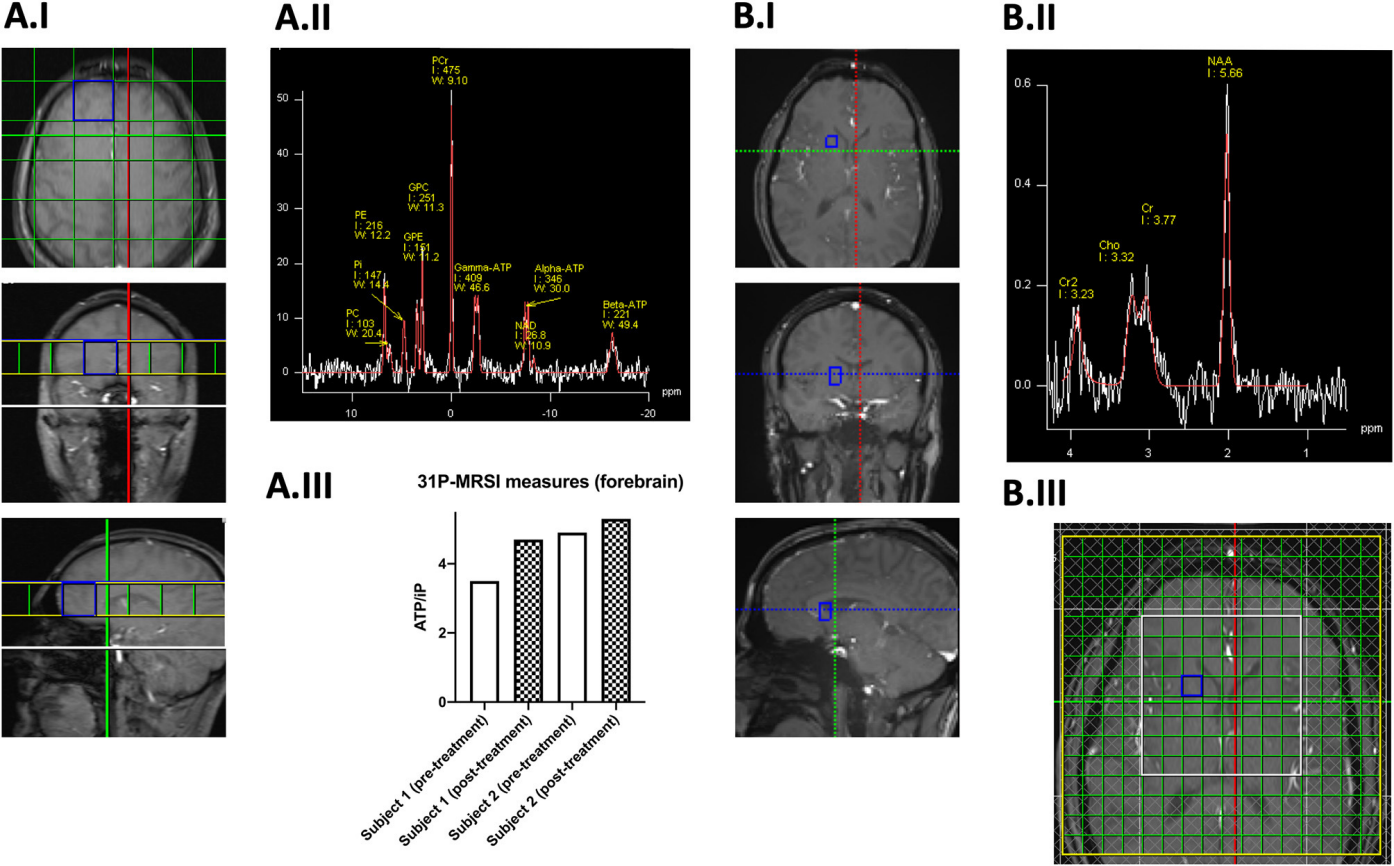
Preliminary 31-Phosphorus **(A)** and Proton **(B)** Magnetic Resonance Spectroscopy Imaging. In Panel (A.I) the FoV and the grid placement of the 31-Phosphorus Magnetic Resonance Spectroscopy Imaging (31P-MRSI) are shown. Highlighted in blue is one exemplary forebrain voxel of a healthy control in which the respective spectrum (white) and line fit (red) are shown in Panel (A.II.) In Panel (A.III), 31P-MRSI measurements in two related (siblings) homozygous *PINK1* mutation carriers before (clear bars) and after three-day treatment with 300 mg MK-7 (checkered bars). Here, we examined the ratios of ATP/iP (as a unitless measure), which will serve as the primary endpoint in this trial and has been used for the sample size estimation. Panel B illustrates the application of Proton-MRSI (1H-MRSI). In Panel (B.I), the investigated voxel is highlighted in blue, and the respective spectrum (white) and line fit (red) are shown in Panel (B.II). As this data is derived from a healthy control subject, no Lactate peak can be seen. In Panel (B.III), the FoV, grid placement, and saturation bars (gray checkered) can be seen. Again, the investigated voxel is highlighted in blue. 1H-MRSI, Magnetic Resonance Spectroscopy Imaging; 31P-MRSI, 31-Phosphorus Magnetic Resonance Spectroscopy Imaging; ATP, adenosine triphosphate; Cho, choline; Cr, creatine; Cr2, methylene protons of creatine; FoV, Field of View; GPC, glycerophosphocholine; GPE, glycerophosphoethanolamine; I, integral; MK-7, long-chain menaquinone 7, vitamin K2; NAA, N-Acetylaspartate; NAD, nicotinamide adeninen dinucleotide; PC, phosphocholine; PCr, phosphocreatinine; PE, phosphoethanolamine; Pi or iP, inorganic phosphate; W, width.

The molecular basis of Parkinson's disease (PD) is complex, and many cellular components and metabolic processes are involved in the development and progression of the disease (1). Mitochondrial dyshomeostasis is a frequently postulated pathophysiological process that contributes to neurodegeneration in sporadic PD (sPD) (2). Although monogenic PD forms are rare, homozygous or compound-heterozygous mutations in *Parkin* and *PINK1* (mitoPD) are described to lead to mitochondrial dyshomeostasis and consecutive bioenergetic deficits (3). However, when tailoring individualized treatment options for PD patients, it is desirable to target gene-specific pathways and pathophysiological hallmarks of the diseased individual (4). Monogenic PD might thus serve as an experimental model as we can pinpoint the genetic defect to specific molecular pathways. Even though the identification of the underlying disease mechanisms in monogenic PD for driving drug development tends to appear more viable, it is crucial to apply the findings from the selected group of mutation carriers to the much larger group of sPD patients. Deep phenotyping of monogenic PD patients, e.g., by neuroimaging of mitoPD patients, might, therefore, help to identify substantial subsets of sPD patients, who may specifically benefit from proposed treatment approaches (5). Besides, pathophysiology-oriented, accompanying diagnostics (i.e., theranostics, a portmanteau describing combined techniques to simultaneously or sequentially diagnose and treat medical conditions) may leverage clinical trial outcomes (6). Theranostics also provides a unique opportunity for adaptive clinical trial designs, where only the most promising study cohorts or participants will be enrolled, and potential medication-related harm will be warded off study participants (7). In this study, we will investigate the potential benefit of MK-7 (long-chain menaquinone 7, MK-7) in mitoPD patients, sPD patients, and healthy individuals. The *PINK1*-deficient biochemical and behavioral phenotype of *Drosophila* flies was rescued by administering MK-4. The observed effects also extended to *Parkin*-deficient flies (see supplementary data of the reference) (8). They found that MK-4 was necessary for transferring electrons within the electron transport chain (ETC) of Drosophila. The proposed mechanism of action was that, similar to ubiquinone, MK-4 serves as an electron carrier and bypasses complex I and II of the ETC via the Q cycle. This resulted in a more efficient adenosine triphosphate (ATP) production helping to maintain physiological levels of ATP *in vivo*. However, presented data suggested that the proposed method of action also extends to longer-chain menaquinones (such as MK-7), which should be used based on their favorable pharmacodynamics and -kinetics in human studies. Experimental data in rodent models, except for toxicity studies, is currently lacking. However, this might also be due to the limited suitability of rodent models for the investigation of monogenic causes of PD (9). 31P-Magnetic resonance spectroscopy imaging (31P-MRSI) offers the possibility to measure ATP and other phosphorus-containing metabolites non-invasively in human brains (10). Thus, 31P-MRSI could be a suitable method for stratifying sPD patients based on their mitochondrial impairment. Also, it might serve to monitor individual treatment responses in all study participants for subsequent studies. In particular, mitoPD patients are expected to show a significant increase in ATP levels after 1 week of MK-7 administration. Besides, we will use other advanced neuroimaging modalities to characterize critical aspects of mitochondrial bioenergetics (see Figure 1) and biomaterial sampling to investigate mitochondrial metabolism. MK-7 has so far not been used as a study medication for the treatment of PD, but for a variety of other indications, e.g., for the treatment of osteoporosis (16). This provides extensive safety data, including post-market surveillance, as a nutritional supplement. The safety profile of MK-7 is exceptionally favorable, and previous studies have shown excellent tolerability (17–20). Before the conceptualization of this trial, we collected pilot data of two homozygous *PINK1* mutation carriers treated with MK-7 (within the scope of compassionate use). We performed 31P-MRSI measurements pre- and post-medication intake. The observed increase in ATP levels (see Figure 2) was later used for sample size estimation of the present study.

### Objectives

1. To show an increase in the cerebral ATP levels in the mitoPD group after 1 week of treatment with MK-7 compared to baseline.
2. To elucidate the interaction of the sPD and HC group affiliation to an increase in the cerebral ATP levels after 1 week of treatment with MK-7 compared to baseline.
3. To determine the role of complimentary neuroimaging markers (see Figure 1) to explain the variance in treatment response among the different groups after 1 week of treatment with MK-7 compared to baseline.

### Trial Design

**Figure 3:**
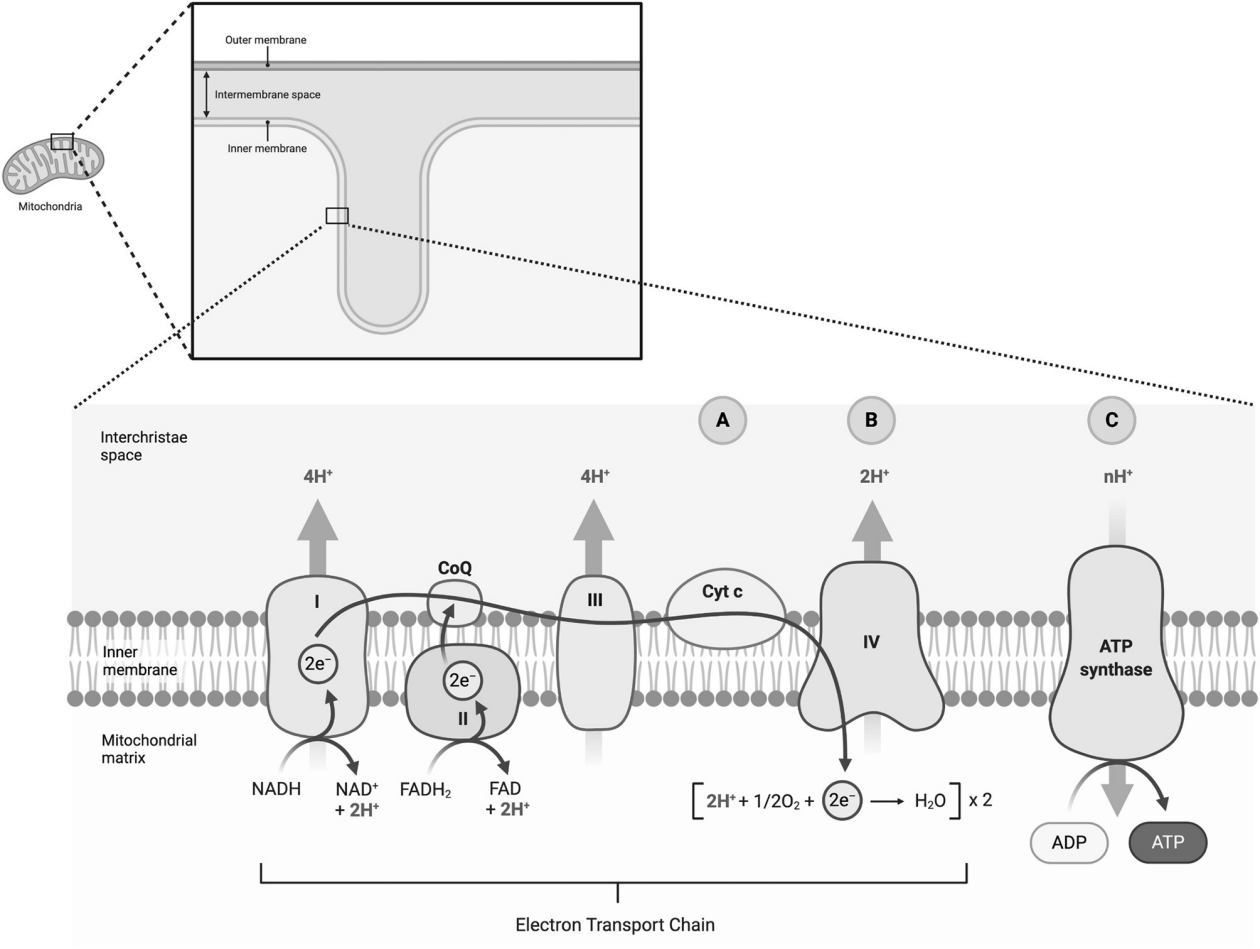
Study groups, sample size, allocation ratio, and intervention assignment. HC, healthy controls; mitoPD, homozygous or compound heterozygous *Parkin* or *PINK1* mutation carrier; MK-7, long-chain menaquinone 7, vitamin K2; sPD, sporadic Parkinson's disease.

The proposed monocentric proof-of-concept study will employ a placebo-controlled, double-blind, and parallel-group design. The allocation ratio of observed study groups will be 1:2:2 (mitoPD, sPD, and HC). A 1:1 randomization to placebo and verum groups (based on age, gender, and disease duration as strata) will be performed for each study group. The sample size estimation was based on available pilot data (see Figure 2). The primary endpoint of this study is defined as the change of the ATP/iP (inorganic phosphate) ratio as measured by 31P-MRSI from baseline visit to the end of the study. We will compare the change of the ATP/iP ratio for each study group (placebo treatment vs. verum), respectively. Hierarchical statistical testing for each study group will be performed (see Figure 3). Secondary endpoints (as stated in Table 1) will be analyzed in an exploratory manner. Metabolic assessments will be considered as potential confounding variables for the variance in cerebral ATP levels at baseline examination. The inclusion and exclusion criteria are based on other studies with MK-7 and the specific requirements for imaging-based endpoints (16, 21). The study is conducted following the regulations of the current ICH-GCP framework (International Council for Harmonization of Technical Requirements for Pharmaceuticals for Human Use, Good Clinical Practice, version E6) (22). Measurements are taken at the beginning and on the 7th day of the intervention. The dopaminergic medication of PD patients (mitoPD and sPD) will be optimized and then kept stable at least 4 weeks before study enrolment. The investigational drug will be administered at a dosage of 1 mg/d. An overview of planned measurements for each study visit is provided in Table 1.

**Table 1:** Overview on study visits and planned examinations.

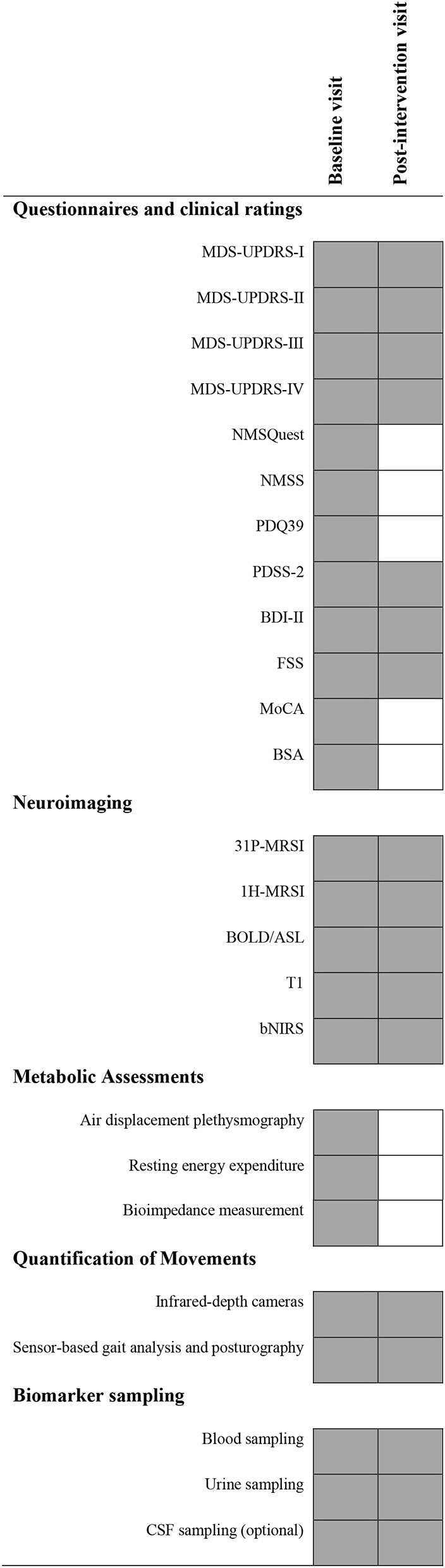

*Darkened fields indicate if stated examination/measurement takes place at the specified visit. In summary, we expect a duration of 4.5 h per study day. The total duration per subject, therefore comes up to approximately 9 h. ASL, Arterial Spin Labeling; BDI-II, Beck's Depression Inventory-II; bNIRS, Broadband Near-Infrared Spectroscopy; BOLD, Blood Oxygen Level Dependent Imaging; BSA, Physical Activity, Exercise, and Sport Questionnaire; CSF, Cerebrospinal Fluid; FSS, Fatigue Severity Scale; MDS-UPDRS, Movement Disorders Society - Unified Parkinson's Disease Rating Scale; MoCA, Montreal Cognitive Assessment; MRSI, Magnetic Resonance Spectroscopy Imaging; NMSQuest, Non-Motor Symptoms Questionnaire; NMSS, Non-Motor Symptom Scale; PDQ39, Parkinson's Disease Questionnaire with 39 items; PDSS-2, Parkinson's Disease Sleep Scale 2*.

## Methods and Analysis

### Methods: Participants, Interventions, and Outcomes

#### Study Setting

The proposed study will be performed at the Department of Neurology of the University Medical Center Schleswig Holstein (UKSH, Campus Lübeck). The UKSH is an academic hospital and the only trial site involved in the conduction of this study. All data will be gathered and stored at this site in accordance with German regulatory policies.

#### Eligibility Criteria

A comprehensive listing of all required inclusion and exclusion criteria can be seen in Table 2. Inclusion criteria follow existing studies and ensure the informed consent of study participants. Exclusion criteria are mainly based on possible differential diagnoses of PD, known potential side effects or drug interactions, as well as general concerns about study participation (e.g., pregnancy).

**Table 2:** Inclusion and exclusion criteria.

**Inclusion criteria**	**Exclusion criteria**
Age >18 years	Comorbidities that might impair informed consent
Written informed consent	MRI contraindications
PD diagnosis based on MDS criteria (for mitoPD and sPD group)	Atypical or secondary parkinsonism
Stable PD medication for 4 weeks prior to enrolment (for mitoPD and sPD group)	Unstable PD medication (if applicable)
	Known allergy/intolerance to MK-7
	Treatment with MK-7, coenzyme Q10, or vitamin K-antagonist 1 month prior to the start of this trial
	Chronic GI malabsorption (e.g., celiac disease, short bowel syndrome)
	Ongoing malignancy (ongoing treatment/clinical controlled visits or diagnosed <5 years ago), excl. NMSC
	Abuse of alcohol or other euphoric drug
	Women who are pregnant or breast-feeding and women who are in the childbearing age without contraception
	Total/subtotal parathyroidectomy
	Treatment with recombined parathormone
	Treatment with bisphosphonates or other anti-osteoporotic drugs
	Treatment with vitamin K antagonists (e.g., warfarine)
	Treatment with statins

*Inclusion and exclusion criteria are based on criteria established in other MK-7 trials to ensure patient safety and the MDS criteria for the diagnosis of PD (23). Additionally, other conditions that might influence the MR-based outcome measures are also considered as exclusion criteria (e.g., history of stroke). GI, gastrointestinal; mitoPD, homozygous or compound heterozygous Parkin or PINK1 mutation carrier; MK-7, long-chain menaquinone 7, vitamin K2; MRI, Magnetic Resonance Imaging; NMSC, Non-melanoma skin cancer; PD, Parkinson's disease; sPD, sporadic Parkinson's disease*.

#### Interventions

Study participants are being randomized to supplementation with MK-7 (1 mg/day, K2VITAL®) or placebo (no active intervention). Study participants will be asked to take the investigated medication once daily for seven days in total. The study tablets (K2VITAL®), including placebo, are provided free of charge by Kappa Bioscience A/S, Silurveien 2B, 0380 Oslo. The company is not involved in the execution of the study or analysis of data. Laboratory testing and drug accountability methods will be applied to verify the participant's compliance. The *in-vivo* metabolism of blood-brain-barrier transport of MK-7 is complex and has not yet been systematically assessed. However, preliminary animal data and our pilot data are highly suggestive for central nervous import and actions (24). To perform drug monitoring and gain deepened pharmacodynamic and -kinetic insights, wewill analyze MK-7 content pre- and postintervention in blood and - for study participants optional–in the cerebrospinal fluid (CSF).

#### Outcomes

The primary endpoint will be the change in the cerebral ATP level measured by 31P-MRSI after 1 week of treatment with MK-7 per group (placebo vs. verum) as a surrogate marker for the treatment of mitochondrial dysfunction in PD patients. We will perform measurements of the hemisphere-averaged forebrain and basal ganglia to determine ATP/iP, PCr/iP, and (ATP+PCr)/iP levels. For the quantification of ATP, the βATP peak of the 31P-MRSI spectrum will be used. For the sake of readability, the manuscript refers to βATP within the scope of 31P-MRSI as ATP. Voxel (30.0 × 30.0 × 30.0 mm^3^) placement is highlighted in Figure 2. Other neuroimaging endpoints will be analyzed on an exploratory basis as no prior pilot data are available for sample size estimations. Additional analyses (i.e., the questionnaires) are meant to characterize the study cohort further and to identify relevant confounders without being considered as endpoints. A concise overview of planned investigations and outcomes is provided in Table 1.

#### MRI Acquisition and Analysis

**31P-MRSI**. 31P-MRSI measurements are performed on a 3T Siemens MAGNETOM Skyra Magnetic Resonance Imaging scanner. A 3T dual tuned quadrature head coil 1H/31P (RAPID Biomedical) is used to acquire the 31P 3D chemical shift imaging (CSI) data with the following protocol parameters: voxel size 30.0 × 30.0 × 30.0 mm^3^, field of view (FoV) 240 × 240 × 240 mm^3^, TR 2,000 ms, TE 2.3 ms, 6-fold weighted averaging, Hamming filtering, flip angle 50°, Nuclear overhauser effect (NOE) disabled (We decided against using NOE to limit the SAR exposure), WALTZ-4 decoupling, total scan time 8 min and 4 s. **1H-MRSI**. 1H-MRSI spectra are recorded with a 64-channel head coil employing a 2D CSI sequence with the following sequence parameters: voxel size 10.0 × 10.0 × 15.0 mm^3^, FoV 160.0 × 160.0 × 15.0 mm^3^, TR 2,300 ms, TE 280 ms, 5-fold averaging, prescan normalization and Hamming filtering, flip angle 90°, water suppression, total scan time 12 min 46 s. Four saturation bands (40 mm thickness, delta frequency−3.60 ppm) are placed around the FoV prior to the measurement of the 1H spectra. **BOLD/ASL**. Previous studies indicated the potential of functional perfusion imaging to study *in vivo* oxygen consumption rates (metabolic rate of oxygen consumption, MRO_2_C) (13, 14, 25). We will perform combined measurements of the BOLD and the cerebral blood flow (CBF) signal with the following scanning parameters: 2D ASL (Siemens product PICORE + Q2TIPS), TR: 2,000 ms; TE: 20 ms; voxel: 4 × 4 × 7 mm^3^ (matrix in-plane 64 × 64); seven slices; Flip angle 75°; TI1: 700 ms; TI2: 1,500 ms; 100 mm thick inversion slab, 15 mm spacing between imaging and inversion slab. We will apply hypo- and hyperventilation challenges for the measurement of MRO_2_C in accordance with the broadband Near-Infrared Spectroscopy bNIRS measurements (see below) (26). MRO_2_C will be determined via a well-established and refined physiological model published by Wise et al. (13). **T1**. Additionally, structural images of the whole brain using a 3D T1-weighted MP-RAGE sequence were acquired (TR = 1,900 ms; TE = 2.44 ms; TI = 900 ms; flip angle 9°; 1 × 1 × 1 mm^3^ resolution; 92 × 256 × 256 mm^3^ field of view; acquisition time 4.33 min, sagittal orientation, phase-encoding direction A>>P) and evaluated by a trained neuroradiologist to rule out the presence of conflicting structural lesions or relevant comorbidities. **Broadband Near-Infrared Spectroscopy**. In addition to MRI-based neuroimaging, we will also perform bNIRS measurements. bNIRS is a system to analyze oxygen utilization via the measurement of the oxidation state of cytochrome-c-oxidase (oxCCO). Besides, it measures changes in cerebral tissue oxygenation and hemodynamics. We will apply well-established hypo- and hyperventilation challenges for the measurement of oxCCO (26).

#### Metabolic Assessments

Body composition will be assessed by air displacement plethysmography (ADP) using the BOD POD system (BOD POD®, Cosmed, Fridolfing, Germany). Body mass will be measured directly before subjects entered the chamber using an electronic scale connected to the BOD POD system. While measurements, subjects are sitting in the chamber wearing tight-fitting underwear and a swim cap. Two repeated measurements of body volume will be performed. If values deviate more than 150 mL, a third body volume measurement will be done. Data will be averaged and body composition parameters, i.e., body fat mass and fat free mass, will be calculated according to manufacturer's software based on Siri's equation for body density (version 5.4.1; BOD POD®, Cosmed, Fridolfing, Germany) including calculated thoracic gas volume. Before each measurement, a two-step calibration will be carried out. Resting oxygen uptake (REE) will be measured by indirect calorimetry using a ventilated hood system for 30 min under standardized conditions in the morning after an overnight fast. During measurement patients will be laying in a supine position awake, quiet and motionless. Body composition will be obtained by multi-frequency bioelectrical impedance analysis (BIA; Nutriguard M, Data Input, Darmstadt, Germany). Tetrapolar BIA measurement of resistance and reactance will be performed at 5, 50 and 100 kHz and 0.8 mA between the wrist and ankle at the dominant site in supine position. Fat free mass (FFM) and fat mass (FM) will be calculated by using the software tool NutriPlus provided by the manufacturer.

#### Questionnaires and Clinical Ratings

To characterize our study group by means of disease severity (if applicable), non-motor signs and symptoms, and daily motor activity, we will apply well-established, validated, and standardized questionnaires and clinical ratings (see Table 1). To add further information to the neurological examination following the established MDS-UPDRS III protocol, we will additionally apply a sensor-based gait analysis to quantify (subtle) changes in motor performance. All MDS-UPDRS III examinations will be videotaped, and a second rating (disregarding rigidity) will be performed on each video in a blinded fashion by an independent movement disorder expert. In case of disagreement of >2 points, a third rater will adjudicate.

#### Quantification of Movements

The clinical examinations will be videotaped, utilizing specialized infrared depth cameras (Motognosis GmBH, Berlin, Germany). This methodology allows us to quantify motor impairment beyond the scope of examiner-driven impressions objectively (27). In addition, we will perform standardized tests of motor function (including instrumented gait and posturography assessments) in accordance to the manufacturer protocol (Mobility Lab, APDM Wearable Technologies, Portland, USA) (28).

#### Participant Timeline

As the proposed study is a short-interventional trial, only two study visits are planned (pre- and postintervention). No up-titration or wash-out phases are scheduled based on the published experiences of other investigators utilizing MK-7. An overview of planned measurements is provided in Table 1.

#### Sample Size

Based on preliminary data, we assume that the strongest effects will be observed in mutation carriers, followed by patients with sporadic PD and healthy controls. In all three groups, the effect of verum and placebo is compared. We will follow a hierarchical test procedure: First, verum and placebo are compared for mitoPD at a significance level of 5%. If the significance level is reached, we will furthermore compare verum and placebo at the same significance level in sPD patients. Finally, if this comparison is significant, we will compare verum and placebo in healthy controls. Preliminary data showed an average increase of 0.925 of the mean ATP/iP ratio under MK-7 treatment in mitoPD, which is assumed as an effect in this study. We continue to assume that no long-term change is observed under placebo and that the standard deviation of the change is about 0.8 according to our pilot data. This leads to an approximate effect size of 1.16. The normality of our sample will be tested via Shapiro-Wilk tests. With a two-sided *t*-test at a significance level of 5% and a statistical power of 80%, *n* = 13 patients must be analyzed in both groups, a total of 26 mutation carriers for this study arm. In the other groups (sPD and HC), a lower effect is expected, which corresponds to an effect size of approximately 0.8. Under the same conditions as above, this corresponds to *n* = 26 patients per group, i.e., 52 sPD subjects and 52 HCs are to be analyzed (see Figure 3).

#### Recruitment

To date, approximately 1,800 samples (PD patients and HCs) have been genotyped and are available for subsequent analysis. We have identified 16 biallelic *Parkin* and *PINK1* mutation carriers and are in contact with those willing to participate in this study (already 61% of the total mitoPD group). We thus anticipate that our recruitment targets are realistic.

### Methods: Assignment of Interventions (For Controlled Trials)

#### Allocation

We have chosen persistent block randomization with stratification for this study. A group of patients will be assigned to a block of study arms and stratified by each configured strata. To ensure that the study is balanced, the block list search filters for strata. Each patient must fit into at least one strata. Age, gender, and disease duration were selected as stratification factors. The categorical characteristic gender has only a few expressions “male,” “female,” etc. and will be stratified directly. We will divide each stratification characteristic into any group. Equal weighting of the study arms within the randomization process will be realized. The randomization will be carried out automatically and independently from the study team by the Clinical Trial Management System (CTMS) after the registration of the subject (29).

#### Blinding

All members of the investigational team will be blinded regarding the type of intervention. As the proposed study employs a double-blind design, neither the members of the study team nor study participants will know whether they receive verum or placebo. Study participants and the investigational team will be unblinded whenever serious health concerns (e.g., SUSARs) arise.

### Methods: Data Collection, Management, and Analysis

#### Data Collection Methods

The study management and the scientists involved are responsible for data processing. The data collection is carried out for the purpose of the research project mentioned above. The study data will be stored and collected electronically. Therefore, services of the IT Center for Clinical Research, Lübeck (ITCR-L) are used. The ITCR-L operates CentraXX, a complete clinical trial management system (CTMS) for structured data collection and storage that provides the foundation for study data storage, reporting and analysis. For the creation of electronic case report forms (eCRFs) and the documentation of studies, the ITCR-L offers an integrated software solution including plausibility checks, data backup, access controls and randomization. With the help of the study data acquisition, the actual study is carried out and the results are documented. For data acquisition eCRFs are used, which can be carried out for the individual probands for the upcoming visit. During and after the study data collection, the principal investigator can view and edit the respective status of all eCRFs available for the study. An audit trial will be established for eCRF entries. An overview of all validation failures that have occurred, which can be directly checked and resolved at this point, is available to the study director at any time. Validation reports and reviews can be executed and result reports can be generated. An automatic transfer of laboratory values for study participants via an HL7 ORU (www.hl7.org) interface to the laboratory system is planned. The investigational team will receive proper training on the eCRF devices.

#### Data Management

The data is stored in a professional CTMS. The CTMS is operated in the demilitarized zone of the University Medical Center Schleswig Holstein. A tight rights-and-role concept protects the data from unauthorized access. During the editing process of the eCRFs, two different CRF statuses (“In process” and “Finalized”) are available. Changes to an already finalized eCRF are only possible through a corresponding right assigned to the user via distinct study roles. The changes are audited. Besides, authorized users within the study data collection can process the release and verification workflows for the eCRFs. An annotation function and the possibility of processing validation failures directly at the CRF is available for the later validation phases after data acquisition. Validation rules include regular expressions (RegEx), ranges, and conditions. No directly identifying characteristics are recorded in the eCRFs, therefore the data is considered pseudonymized. A mobile app is used to survey the eCRFs. The mobile devices are equipped with a kiosk mode software protecting them from improper use. The kiosk mode prevents all use of hardware functions and only allows the use of the app for study data documentation. The data acquisition on the tablets is entirely offline. Only for data transfer, a connection to the server is required. The transmission can be made either via the USB-LAN adapter or via a secured WiFi. After data entry, the completed questionnaire (encrypted via SSL/RPC) is sent to the server. After successful transmission, the eCRF data on the tablet is automatically deleted.

#### Statistical Methods

The statistical approach on the primary study endpoint has already been addressed in the paragraph about sample size estimation. The analysis will be performed following the intention-to-treat methodology (ITT). All secondary endpoints will be reported as descriptive statistics or as exploratory analyses (see Objective 3).

### Methods: Monitoring

#### Data Monitoring

Although safety monitoring is an essential part of any study, a Data Monitoring Committee (DSMB) is not required due to the following reasons. The establishment of a DSMB can be vital for studies that involve: (I) Saving lives, (II) the monitoring of safety in long-term studies, including in the case of non-life-threatening diseases, or (III) the prevention of severe damage to health. As none of the conditions mentioned above apply to the proposed study, and we do not expect any severe harm for our study participants based on established knowledge (see below), we refrained from establishing a DSMB.

#### Harms

MK-7 has already been used in several preclinical and clinical studies (16, 17, 21). Doses of 1 g/d have already been used here for more extended periods than in this study without any significant risks for the subjects (17). Due to the excellent tolerability of the investigational product, no dosing phase is required. Some reports mentioned the occurrence of diarrhea and nausea; since many PD patients are dependent on medication to stimulate digestion, the side effects might be counteracted by reducing this medication (18). The analysis of blood coagulation was included in the safety laboratory, along with the study of liver and kidney values. For MK-7, no disruption of the hemostasis or the liver and kidney function are described. But since the substance is metabolized, excreted, and the role of MK-7 in coagulation processes is well-known, harm for participants is at least theoretically possible while applying high dosages. Besides, the study participants receive medications for treating the symptoms of PD, which will be continued and kept stable during the interventional period. Potential physiological or medication-related interactions cannot be ruled out in advance, which is why laboratory controls, as well as close interviews on possible side effects, are planned for safety reasons. However, no such interactions were yet described from previous studies in comparable cohorts. Overall, a favorable risk-benefit ratio can be assumed. Study participants will be interviewed regarding any side effects or (S)AEs. We will provide standardized (S)AE reportings and possibilities for immediate unblinding. To detect subclinical impairments of health, we will conduct safety laboratory measures, addressing hematological, nephrological, and gastroenterological side effects.

#### Auditing

Study auditing will be performed via independent colleagues of the investigational team following the recommendations of the Medicines and Healthcare Products Regulatory Agency (MHRA) and the respective GCP guidelines (22, 30).

## Discussion

The present project offers an innovative, translational approach that investigates a well-tolerated investigational drug as a short intervention (based on promising preliminary experimental data) in PD.

An important reason for the failure of earlier clinical trials in PD research may be that the recruitment of patients in these studies was based on purely clinical criteria (4). Biological pathway-oriented markers might help to avoid treating a heterogeneous patient collective with different underlying disease-causing mechanisms (5). Homozygous or compound heterozygous point mutations, as well as deletions and multiplications in the genes *Parkin* and *PINK1*, are the most common cause of early-onset recessive inherited PD (31). Besides, there is sound evidence that mitochondrial dysfunction is also present in a relevant proportion of sPD contributing to the pathophysiology (2). Thus, therapeutic options for the rare monogenic forms with mutations in *Parkin* and *PINK1* could also be helpful for a larger group of sPD patients fulfilling such a biomarker profile. These observations form the basis of the proposed study. We are not only going to see differences of *in vivo* mitochondrial dysfunction but aim to also identify individual predictors of treatment response (32). The latter will substantially help to design future trials more efficiently (e.g., adaptive study design for neuroprotective trials engaging more extended interventional periods). MK-7 might serve as a “mitochondrial enhancers,” through supporting electron transport within the ETC, and is, therefore, suitable to validate our neuroimaging approach (8). This study would be an essential step to elucidate at least one therapeutically relevant disease mechanism for a substantial subset of PD patients. Additionally, it would be a first step to successfully make treatment decisions based on the genetic status of PD patients and translate progress in molecular genetics into personalized patient care (4). The individualized therapy of preselected patients and the use of pathophysiologically oriented endpoints may provide the basis for subsequent clinical studies (with extended intervention periods) to investigate possible neuroprotective aspects of improving *in-vivo* mitochondrial bioenergetics. If this proof-of-principle study is successful, future questions will include: Is the benefit seen sustained, is it a mere symptomatic effect associated with improved energy metabolism or can it indeed exert neuroprotective effects? Does the effect of MK-7 also extend to heterozygous *PINK1/Parkin* mutation carriers affected by PD? More extensive trials and long-term follow-ups would then be warranted. Identifying predictors of treatment response in sPD patients would also be of great interest, e.g., based on complex-genetic considerations, to additionally identify subgroups of PD patients with a distinct mitochondrial dysfunction. This would imply that this treatment option would no longer be restricted to monogenic alterations of mitochondria-related genes and could, therefore, result in a more significant impact on improving PD patient care.

## Ethics and Dissemination

### Research Ethics Approval

The proposed trial has been approved by the responsible, independent ethics committee Lübeck before the start of the trial (docket number AZ19-320). Participation in the study is voluntary. Investigators inform patients of risks and benefits, their rights and burden. Written informed consent in the study will be obtained from the patients personally before any trial-related intervention. Refusal to participate will not affect the following patient care. The study will be conducted in accordance with the Declaration of Helsinki and the requirements of all applicable local and international standards, e.g., data protection law (privacy of data is secured by collection of pseudonymized data).

### Protocol Amendments

Currently, no protocol amendments are planned. In the case of protocol amendments becoming necessary, the ethics committee in Lübeck will be the responsible authority. If substantial protocol amendments are granted, the study protocol will be updated in the German Clinical Trial Registry DRKS, DRKS00019932.

### Consent or Assent

As one of our inclusion criteria states that only individuals with the age of 18 years or above might be enrolled, assent does not apply to this study. Informed consent will be obtained by the study physician. The consent forms for the collection of biomaterials were conceptualized in accordance with the Association of Medical Ethics Committees in Germany (https://ak-med-ethik-komm.de/index.php?option=com_content&view=featured&Itemid=458&lang=en, date of access: 27th of February 2020).

### Confidentiality

All employees are bound to secrecy. The data is protected from unauthorized access. The collected data will be anonymized after reaching the study objective, but at the latest after 10 years, unless more extended archiving periods are stipulated by law. Participants are comprehensively informed about their rights (e.g., deletion) within the framework of the General Data Protection Regulation (GDPR) of the European Union (33). Also, the right of complaint to the responsible data protection supervisory authority is referred to in the subject information.

### Access to Data

Only employees of the study have access to gathered data. Other research groups might apply for access to study data depending on whether participants have consented in the sharing of anonymized data, as stated in their consent forms. Data sharing will be considered after completion of the study on reasonable request.

### Ancillary and Post-trial Care

Post-trial care will be performed within the framework of general medical care. In case of unlekikely harm, the study participants are insured via the UKSH.

### Dissemination Policy

Study results will be published open access in a peer-review journal and will be registered in relevant databases additionally. The Parkinson's Foundation (Miami, US), as the principal founder of this trial, will actively perform public engagement and will use their respective distribution channels (e.g., mailing lists).

## Ethics Statement

The proposed studies involving human participants were reviewed and approved by the responsible, independent ethics committee Lübeck (docket number AZ19-320). Participants will provide their written informed consent to participate in the study prior to trial-related intervention.

## Author Contributions

JP: conception, organization, and execution of the research project, design, execution, and review and critique of the statistical preparation, and writing the first draft and review and critique of the manuscript. MK: conception, organization, and execution of the research project, execution of the statistical preparation, and review and critique of the manuscript. MV: conception of the research project, execution of the statistical preparation, and review and critique of the manuscript. IK: design and execution of the statistical preparation, and review and critique of the manuscript. SMS and BW: conception, organization, and execution of the research project and review and critique of the statistical preparation and review and critique of the manuscript. CK and NB: conception, organization, and execution of the research project, review and critique of the statistical preparation, and review and critique of the manuscript. All authors contributed to the article and approved the submitted version.

## Conflict of Interest

The authors declare that the research was conducted in the absence of any commercial or financial relationships that could be construed as a potential conflict of interest. The handling editor declared a past co-authorship with one of the authors CK.
